# Development of an innovative approach using portable eye tracking to assist ADHD screening: a machine learning study

**DOI:** 10.3389/fpsyt.2024.1337595

**Published:** 2024-02-15

**Authors:** Jae Hyun Yoo, ChangSu Kang, Joon Shik Lim, Bohyun Wang, Chi-Hyun Choi, Hyunchan Hwang, Doug Hyun Han, Hyungjun Kim, Hosang Cheon, Jae-Won Kim

**Affiliations:** ^1^ Department of Psychiatry, Seoul St. Mary’s Hospital, College of Medicine, The Catholic University of Korea, Seoul, Republic of Korea; ^2^ Department of Computer Science, Gachon University, Seongnam-si, Gyeonggi-do, Republic of Korea; ^3^ Department of Psychiatry, Seoul Metropolitan Government - Seoul National University (SMG-SNU) Boramae Medical Center, Seoul, Republic of Korea; ^4^ Department of Psychiatry, Chung-Ang University College of Medicine, Seoul, Republic of Korea; ^5^ AI.ble Therapeutics, Seoul, Republic of Korea; ^6^ Division of Child and Adolescent Psychiatry, Department of Neuropsychiatry, Seoul National University Hospital, Seoul, Republic of Korea

**Keywords:** attention-deficit/hyperactivity disorder, eye-tracking technology, saccades, fixation, biomarkers, machine learning

## Abstract

**Introduction:**

Attention-deficit/hyperactivity disorder (ADHD) affects a significant proportion of the pediatric population, making early detection crucial for effective intervention. Eye movements are controlled by brain regions associated with neuropsychological functions, such as selective attention, response inhibition, and working memory, and their deficits are related to the core characteristics of ADHD. Herein, we aimed to develop a screening model for ADHD using machine learning (ML) and eye-tracking features from tasks that reflect neuropsychological deficits in ADHD.

**Methods:**

Fifty-six children (mean age 8.38 ± 1.58, 45 males) diagnosed with ADHD based on the Diagnostic and Statistical Manual of Mental Disorders, fifth edition were recruited along with seventy-nine typically developing children (TDC) (mean age 8.80 ± 1.82, 33 males). Eye-tracking data were collected using a digital device during the performance of five behavioral tasks measuring selective attention, working memory, and response inhibition (pro-saccade task, anti-saccade task, memory-guided saccade task, change detection task, and Stroop task). ML was employed to select relevant eye-tracking features for ADHD, and to subsequently construct an optimal model classifying ADHD from TDC.

**Results:**

We identified 33 eye-tracking features in the five tasks with the potential to distinguish children with ADHD from TDC. Participants with ADHD showed increased saccade latency and degree, and shorter fixation time in eye-tracking tasks. A soft voting model integrating extra tree and random forest classifiers demonstrated high accuracy (76.3%) at identifying ADHD using eye-tracking features alone. A comparison of the model using only eye-tracking features with models using the Advanced Test of Attention or Stroop test showed no significant difference in the area under the curve (AUC) (p = 0.419 and p=0.235, respectively). Combining demographic, behavioral, and clinical data with eye-tracking features improved accuracy, but did not significantly alter the AUC (p=0.208).

**Discussion:**

Our study suggests that eye-tracking features hold promise as ADHD screening tools, even when obtained using a simple digital device. The current findings emphasize that eye-tracking features could be reliable indicators of impaired neurobiological functioning in individuals with ADHD. To enhance utility as a screening tool, future research should be conducted with a larger sample of participants with a more balanced gender ratio.

## Introduction

Attention-deficit/hyperactivity disorder (ADHD) is a neurodevelopmental disorder that affects 3–10% of children (1). Symptoms of ADHD, such as inattention, hyperactivity, and impulsivity significantly impair social, academic, and occupational functions, and can even persist into adulthood (2). As such, early detection and intervention are crucial for the recovery of patients with ADHD.

Currently, the diagnosis of ADHD relies on expert decisions informed by reports from parents’ and/or teachers’, behavioral observations, and clinical interviews (3). Several task-based paradigms have been developed and are widely applied in both clinics and research to assess attention and executive dysfunction in individuals with ADHD. These include the Continuous Performance Test (CPT) (4), Tests of Variables of Attention (T.O.V.A.) (5), and neuropsychological batteries such as the Stroop test (6) and the Wisconsin Card Sorting test (7). However, task-based measures for diagnosing ADHD have pitfalls, including a high false positive rate, limited test–retest reliability, and practice effects (8). In clinical settings, physicians employ a multifaceted assessment considering additional factors such as recent stress, anxiety, depression, and behavioral problems that can influence attention (9). However, physician interviews are not suitable for screening large populations of children as they are time-consuming and expensive.

Recently, significant attention has been paid to the characteristics of eye movement and their neurobiological roles in ADHD (10–13). Eye movements are controlled by complex brain regions, including the frontal eye field, dorsolateral prefrontal cortex (DLPFC), posterior parietal cortex, basal ganglia, and cerebellum (14, 15). There is significant overlap between the neural networks involved in oculomotor and attention control (16), and there is evidence to suggest that the DLPFC and substantia nigra pars reticulata likely provide essential control signals for saccadic suppression (17, 18). Experimental inhibition in these areas leads to increased intrusive saccades, similar to deficits seen in ADHD, suggesting an alteration in frontostriatal circuitry affecting suppression signals crucial for saccadic control in ADHD individuals (19). These findings indicate that eye movement features could be potential biomarkers for cognitive processes, including those related to attention and brain function.

Eye-tracking studies in children with ADHD have shown considerable potential in discriminating children with ADHD from typically developing children (TDC). In a meta-analysis, lower performance in direction errors in the anti-saccade task and visually guided saccade latency was identified in the ADHD group compared to the TDC group (16). Another study demonstrated a relationship between premature anticipatory eye movements and the inattentive characteristics associated with ADHD (20). In addition, the ADHD group showed greater errors (21, 22) and a longer latency in the memory-guided saccade task, as well as more eye movement during the fixation task (23). However, these findings have limited generalizability because of the limited sample size, lack of a uniform task paradigm, and heterogeneity in the results among studies (20–22). While the findings from these studies remain inconclusive, eye-tracking holds promise as a potentially valuable tool for screening ADHD based on neurobiological markers.

In the present study, we aimed to develop a model that best distinguishes children with ADHD from TDC through the application eye-tracking features. We used machine learning (ML) to combine features extracted from five different eye-tracking tasks that require attentional control, working memory, and response inhibition, which are crucial neuropsychological impairments in ADHD (24). Furthermore, we measured the feasibility of the eye-tracking-features-only model and its potential as a screening tool for ADHD. For this purpose, we estimated the classification performance of the eye-tracking-features-only model (stage 1), and compared it with that of the conventional screening methods; Advanced Test of Attention (ATA) and/or Stroop task (stage 2). Finally, we measured the performance of a model that combined demographic, behavioral, and clinical information on ADHD obtained from physicians’ examinations and contrasted its performance with that of the stage 1 model (stage 3).

## Methods

### Participants

We recruited children aged 6–12 years through advertisements at four university hospitals and one elementary school in Seoul, Korea. Participants voluntarily visited one of the hospitals and underwent clinical and semi-structured diagnostic interviews using the Korean version of the Kiddie-Schedule for Affective Disorders and Schizophrenia for School-Age Children-Present and Lifetime Version (K-SADS-PL-K) (25) by a child and adolescent psychiatrist. Based on these interviews, a diagnosis of ADHD was determined according to the ADHD criteria of the Diagnostic and Statistical Manual of Mental Disorders, Fifth Edition (DSM-5). Individuals who did not meet any DSM-5 criteria, had no history of psychiatric disorder in first degree relatives, and were not taking any medication that could affect the nervous system (including psychiatric medication and anticonvulsants) were classified as TDC. Additionally, intelligence quotient (IQ) was measured for each participant using the Korean Wechsler Intelligence Scale for Children-Fourth Edition and Fifth Edition.

The exclusion criteria were as follows: (1) any history of medical or neurological disorders; (2) IQ < 70; and (3) any history of autism spectrum disorder, psychotic, bipolar, or eating disorders.

### Ethical statements

This study was approved by the Institutional Review Boards of Seoul National University (SNU) Hospital (Approval Number 2103-197-1208), Chung-Ang University Hospital (2160-003-464), Seoul St. Mary’s Hospital (KC21FIDI0355), and Seoul Metropolitan Government (SMG)–SNU Boramae Medical Center (30-2021-111). Written informed consent was obtained from all participants and their caregivers. The study protocol was approved by the Institutional Committee on Human Research, and conformed to the ethical guidelines of the 1975 Declaration of Helsinki.

### Clinical symptom assessment

After enrollment, the severity of ADHD symptom was determined by one of the participants’ caregivers using the Korean version of Dupaul’s ADHD Rating Scale IV (ADHD-RS) (26). The Child Behavior Checklist (CBCL) (27) and the Disruptive Behavior Disorders Rating Scale (DBDRS) (28) were obtained from the primary caregivers of the participants to estimate the children’s levels of internalizing and externalizing symptoms.

Participants were asked to complete questionnaires including the Children’s Depression Inventory (CDI) (29) and the Beck Depression Inventory (BDI) (30, 31). The Screen for Child Anxiety Related Disorders (SCARED) (32, 33), and Family Adaptability and Cohesion Evaluation Scale IV (FACES-IV) (34) were further applied to assess subjective distress related to depression, anxiety, and family functioning, respectively. Finally, the Stroop task and the Auditory/Visual Continuous Performance Test (ATA) (35) were administered to each participant. In the Stroop task (6), four representative T-scores (word, color, color-word, and interference scores) were measured. Similarly, the T-scores of omission error (OE), commission error (CE), response time (RT), and variability in RT were computed from each visual and auditory ATA. T-score data (four scores from the Stroop task and eight scores from the ATA) were included as behavioral task features in the ML analysis.

### Eye-tracking experiment and behavioral paradigms

The eye-tracking system used for the experiment comprised a visual display, a connecting server, and a main server computer for deep learning. The tasks were initially recorded with a camera lens attached to the Android 10 system built into the Samsung Galaxy Tab 7+. SeeSo SDK (built on Unity SDK 2.4.2), developed by VisualCamp, was used to capture frontal facial images and to calculate 2D gaze points. Digitized data were stored on each mobile pad and subsequently transferred to the main server computer.

During the experiment, the participants were asked to sit in an upright position, and their chairs were height-adjusted so that the participant’s face was at the center of the smart pad device. The screen was placed approximately 50 cm away from the participants’ faces. Before performing the behavioral tasks, each participant was asked to calibrate the eye-tracking system to accurately estimate their eye movements. To become accustomed to eye rolling while minimizing head movement, participants were subjected to five practice stimuli before the initiation of each task.

We excluded eye-tracking data if the x- and y-coordinates did not follow the gaze and remained stalled at a fixed coordinate, or if the gaze was tracked, but the reference point of the x- or y-coordinate was off-screen.

**Figure 1:**
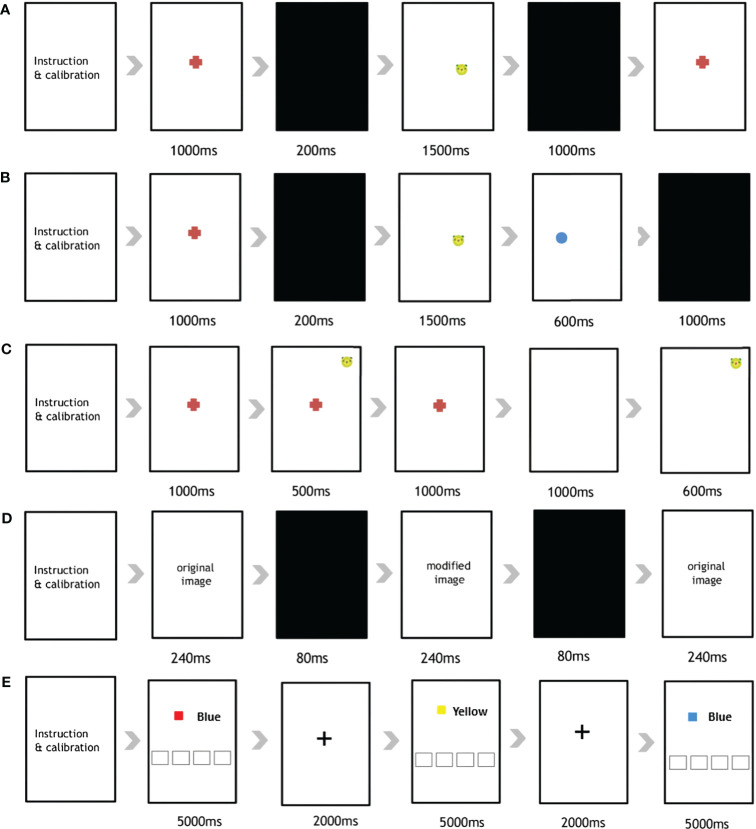
Overview of the five eye-tracking tasks used in the current study. The **(A)** Pro-Saccades Task, **(B)** Anti-Saccades Task, **(C)** Memory Guided-Saccades Task, **(D)** Change Detection Task, and **(E)** Stroop Task.

The eye tracking experiment comprised five serial tasks (**Figure 1**): (1) Pro-Saccades Task (PST), (2) Anti-Saccades Task (AST), (3) Memory-Guided Saccades Task (MGST), (4) Change Detection Task (CDT), and (5) Stroop Task.

First, the PST was administered to participants using a previously described protocol (12). The PST is a visually guided saccade task comprising 30 stimuli (**Supplementary Figure 1**). The participants were all given specific instructions to initially focus their gaze on a central fixation point (FP). Subsequently, they were required to shift their gaze from the FP location to a peripheral target stimulus (animal face illustration), which unpredictably appeared on either the left or right side of the smart pad device. After an additional 1,000 ms, one of two scenarios unfolded; either the FP vanished, and after a gap duration of 200 ms, an eccentric target stimulus emerged; or, the target stimulus remained illuminated for 1,000 ms.

In the AST (**Supplementary Figure 2**), the presentation of the stimuli mirrored the previously described procedure (12). Participants were instructed to direct their gaze toward the central FP. However, upon the appearance of the eccentric stimulus, they were directed to shift their gaze away from the stimulus and toward the opposite side of the vertical meridian. The stimulus location was assigned randomly to either side. This task comprised 48 anti-saccade trials 5 seconds apart.

The MGST was administered to assess visuospatial working memory. The MGST procedure followed that indicated in the original article (36, 37), with the following time adjustments (**Supplementary Figure 3**). The participants were initially asked to gaze at the FP for 1,000 ms, and the target stimulus appeared at the peripheral region of the screen (500ms) according to randomly assign order, then disappeared (**Supplementary Figure 2**). After the disappearance of the FP (1,000ms), participants needed to use their memory to direct their eyes toward a remembered location in the absence of a visual stimulus. The stimulus location was randomized over trials so that the participant could not predict where the cue would appear on any given trial. A total of 36 trials were included in the MGST.

The CDT is designed to assess visuospatial working memory and attention capacity. The procedure for the CDT was the same as that in the original paper, using the same time schedule (**Supplementary Figure 4**) (38, 39). A total 15 pairs of images was used, with each pair displaying minor differences in color, location and presence/absence at the center or periphery. The first image was displayed for 240ms, after which it was changed to another pair of images sequentially (240ms), with a black background (80ms) in between. Participants were asked to identify the differences between images to examine their visual working memory. In our application, we made a circle appear when participants touched the screen. This way, they knew what they had chosen. If the subject answered correctly, they moved to the next image pair; if not, they moved to the next pair after 20 seconds.

Finally, the Stroop task was implemented to assess response inhibition and working memory when processing incongruent stimuli (40). For this, we employed the Stroop paradigm (**Supplementary Figure 5**), as utilized by Vakil et al. (41). One of the 4 color name were exhibited using “Malgun Gothic” font, black-colored, size 144, while the colors themselves were displayed within a rectangle. Participants were instructed to identify the color within the rectangle by pressing the corresponding name on the lower side of screen. The task comprised a total of 40 trials, including 50 congruent and 50 incongruent trials. After a stimulus appeared on the screen (5,000ms), 2,000 ms of FP was followed as a signal that the trial had changed. If the subject answered correctly, they moved to the next trial; otherwise, a gap of after 5 seconds was included before moving on.

### Selection of eye-tracking features and identification of the optimal model using ML algorithms

From each task, we extracted 20 distinct eye-tracking features (**Table 1**). In the current experiment, a fixation was defined as a cluster of points where the distance between points indicated they were spatially close to each other, and the temporal interval was longer than 70 ms. The saccade was defined as an eye gaze movement from one point to another. We estimated the degree and latency of each saccade and elicited the total degree and its average and standard deviation as features. Additionally, the total count, total duration, mean duration, and longest time spent on saccades and fixations (Saccade Time Max, and Fixation Time Max) were obtained. To estimate the extent of the participants’ attention, the total and mean time spent looking at the screen, hit count, and total elapsed time on the task were recorded as eye-tracking features. Finally, the average x and y coordinate values of the participant’s gaze were calculated from the eye-tracking data.

**Table 1:** Twenty eye-tracking features extracted from each of five sequential tasks.

Features	
Saccade Degree Mean	Fixation Count
Saccade Degree SD	Fixation Mean
Saccade Degree Total	Fixation Duration
Saccade Latency Mean	Fixation Time Max
Saccade Latency SD	Coordinate X Mean
Saccade Latency Total	Coordinate Y Mean
Saccade Count	Screen Duration Mean
Saccade Mean	Screen Duration Total
Saccade Duration	Hit Count
Saccade Time Max	Total Elapsed Time

Feature selection is essential to reduce the influence of features of low importance, avoid over-fitting, and to improve the generalization of the model (42). In a comparative study of multiple ML algorithms, feature selection using RandomForest showed the best performance among a variety of datasets (42). Thus, we employed the RandomForest classifier from the python ‘scikit-learn’ library for feature selection across the five serial tasks. The accuracy was measured using the 5-fold cross-validation (CV) method, and the rank of each feature was determined based on the feature importance metric, calculated using the ML algorithm. For each fold of the group, 2000 iterative tree generation processes were performed to establish a stable estimation model. Subsequently, the backward elimination method was employed to remove features with the lowest importance value and classification accuracy of the model. Once the features with less importance were removed, the feature set demonstrating the highest averaged 5-fold accuracy was identified for each task.

Next, we tested the estimation power of the selected eye-tracking features subset using various ML algorithms built into the ‘scikit-learn’ library. A model with an accuracy of 0.1 or greater than the Dummy classifier (a model that classifies all participants as TDC), was determined as a model with superior classification power. Soft voting is a helpful technique that uses an average of the probabilities between the predictions of multiple classifiers to determine the final class (43). To enhance flexibility and generalization of our classification model, we combined the superior models (RandomForest classifiers and Extra Trees classifires) using soft voting methods. Each model included in the soft voting methods was tuned to the value which showed the highest accuracy through hyperparameter analysis.

Finally, we examined the utility of the eye-tracking-features-only model as a screening measure. We considered AUC (separability) as an important metric, along with Recall (sensitivity), to determine its potential as a screening tool. The classification performance (Accuracy, Recall, Precision, and F1 score) of each model was determined using the eye-tracking feature only model (Stage 1), conventional ADHD screening tools (ATA, Stroop task, and both) (Stage 2), and optimal diagnosis using combined demographic, behavioral, and clinical information (Stage 3). To verify the value of the eye-tracking-only model as a screening tool, the area under the curve AUC was obtained and compared to compare the receiver operating characteristics (ROC) of the models at each stage.

The features included in each stage were as follows:

Stage 1: Eye-tracking features only [33 features in total].
Stage 2: Visual and auditory ATA only [8 features]; Stroop task only [4 features]; both ATAs and Stroop task [12 features in total].
Stage 3: Eye-tracking features [33 features], demographic features (age, sex, IQ), behavioral task features (features from the both ATAs [8 features] and the Stroop task [4 features]), and clinical characteristics (CBCL [13 features], DBDRS [3 features], CDI, BDI, SCARED [6 features], and FACES-IV [3 features]) [75 features in total].

### Statistical tests

The demographic and clinical characteristics, ADHD symptoms, and behavioral task performance of the ADHD and TDC groups were compared using parametric (independent-sample t-tests) and non-parametric (Mann-Whitney U test) tests, depending on whether the normality assumption was met. If the p-value of the Kolmogorov-Smirnov test and the Shapiro-Wilk test was greater than 0.05, we determined the data was normal. For categorical variables, we used the chi-square test. Pearson’s correlation was conducted between eye-tracking features and scores of ADHD-RS, ATA, Stroop task. Statistical results were analyzed using SPSS 24.0 software (IBM Corporation, Armonk, NY, USA).

Second, ML analysis of eye tracking and relevant features was performed using Python 3.7.11 and scikit-learn 0.23.2. For the comparison of overall performance of models in each stage, the AUC was measured from each model at different stages. Then, the Wilcoxon rank sum test was performed to compare the AUCs between the models and the model with the eye tracking feature only (Stage 1).

A test result was considered statistically significant if the p-value was < 0.05.

## Results

### Participants and clinical characteristics

**Figure 2:**
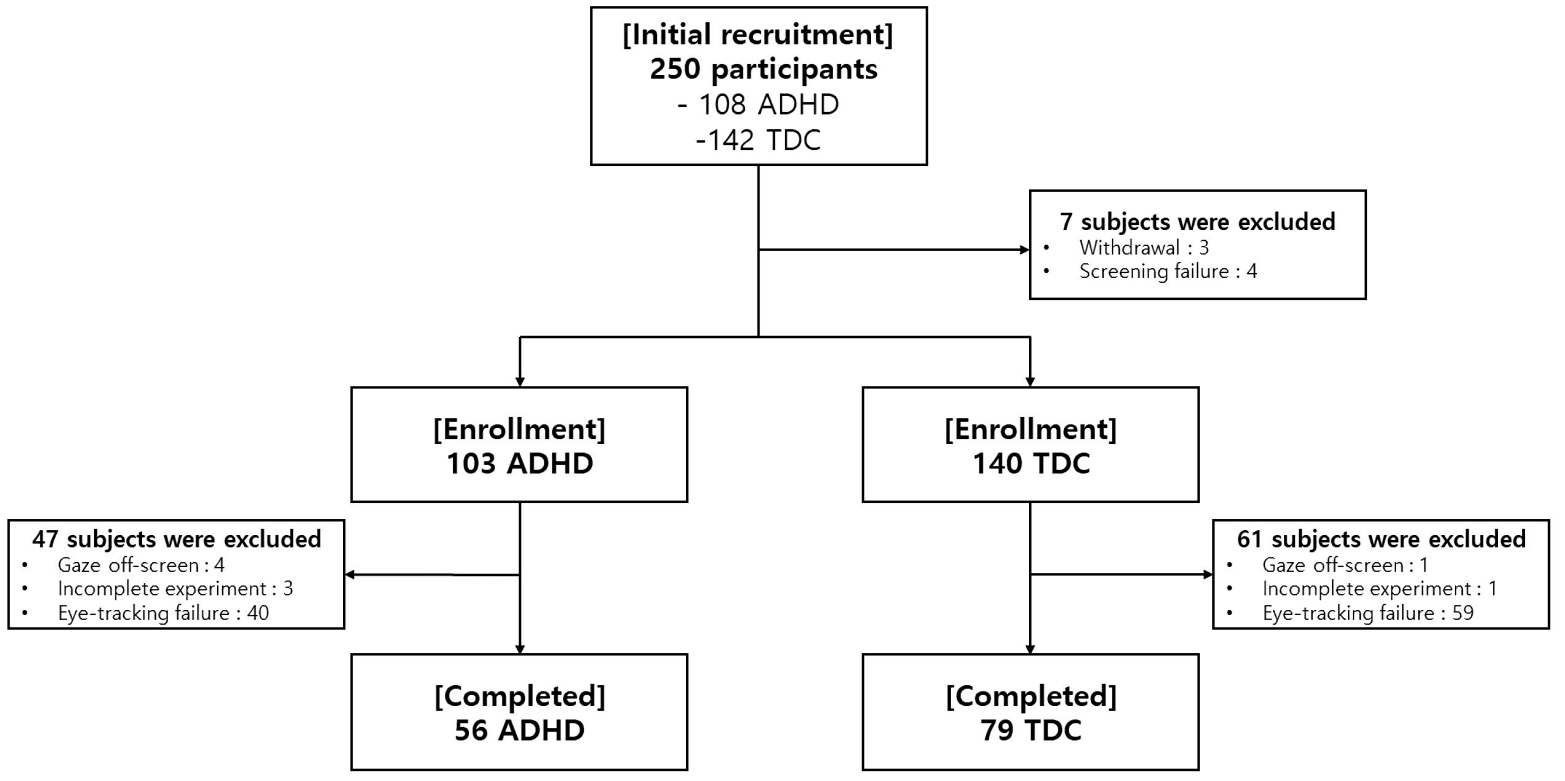
A flowchart showing enrollment of the study participants.

This multicenter study initially recruited 250 participants (108 children diagnosed with ADHD and 142 TDC). Among them, 7 subjects were excluded prior to enrollment (3 withdrawal, 4 screening failure). Further, 47 ADHD children and 61 TDC were excluded due to errors in collecting eye-tracking data (5 Gaze off-screen, 4 Incomplete Experiment, 99 Eye-tracking failure). Finally, 135 participants (56 subjects with ADHD and 79 TDC) completed the eye-tracking experiments (**Figure 2**). From among the 135 participants, the complete sets of clinical characteristics (IQ, CBCL, DBDRS, CDI, BDI, SCARED, and FACES-IV) and behavioral assessments (the visual and auditory ATA and the Stroop task) were collected from 73 subjects (50 with ADHD and 23 TDC).

The ages of participants in the ADHD and TDC groups did not differ significantly (**Table 2**). However, the ADHD group a higher proportion of boys than the TDC group (p<0.001). The average ADHD-RS total score was 25.98 ± 7.81 among the participants diagnosed with ADHD, which is higher than the clinical cutoff value (19 and above). Significant differences were observed in the ADHD-RS total scores (p<0.001), inattention (p<0.001), and hyperactivity/impulsivity domain scores (p<0.001) between the two groups.

**Table 2:** Demographics of all study participants (n=135).

Variables	ADHD (n=56)	TDC (n=79)	P value
Age, years †	8.38 ± 1.58	8.80 ± 1.82	.053
Gender (M:F)	45:11	33:46	<0.001
ADHD-RS total score †	26.27 ± 8.46	9.77 ± 5.71	<0.001
ADHD-RS inattention score †	14.88 ± 4.82	6.16 ± 3.87	<0.001
ADHD-RS hyperactivity/ impulsivity score †	11.39 ± 5.31	3.61 ± 2.62	<0.001

ADHD, Attention-Deficit/Hyperacitivity Disorder; TDC, Typically Developing Children; ADHD-RS, Dupaul’s ADHD rating Scale, Korean version.

†Mann-Whitney U test was applied because of violations of the normality assumption.

A comparison of the behavioral task measurements revealed that participants diagnosed with ADHD had higher OE and CE production in the visual and auditory ATA, as well as lower verbal fluency and inhibitory control performance in the Stroop task, although their average IQ score did not significantly differ from that of the control group (p = 0.770, **Supplementary Table 1**).

Self-administered questionnaires showed that the ADHD group had higher levels of depressive and anxiety symptoms than the TDC group. Primary caregivers also reported that participants diagnosed with ADHD had more externalizing problems (includes Delinquent and Aggressive Behaviors), internalizing problems (includes Withdrawn, Anxious/Depressed), and total problems (includes Social, Thought, and Attention problems) in the CBCL, and disruptive behaviors in the DBDRS scale (**Supplementary Table 1**).

Correlation results of eye-tracking features and conventional screening methods (includes scores of ADHD-RS, Stroop task, and visual and auditory ATA), are summarized in online **Supplementary Tables 2, 3**.

### Classifying ADHD group from TDC using eye-tracking features only

After recursive feature selection, 33 eye-tracking features from five tasks were retained. The list of selected eye-tracking features and their ranks is displayed in **Table 3**. Before applying the ML algorithm, we constructed an optimal eye-tracking features model by combining all eye-tracking features.

**Table 3:** Comparison of the eye-tracking features between the ADHD and TDC groups, selected from recursive feature elimination.

Task sequence	Feature	Feature Importance	ADHD (n=56)	TDC (n=79)	p-value
Pro-saccade task	Fixation Duration†	0.038	64.57 ± 19.70	71.79 ± 16.90	0.030
	Saccade Degree Mean	0.025	39.13 ± 7.23	37.33 ± 8.20	0.189
	Saccade Latency Mean†	0.021	1.21 ± 0.75	1.08 ± 0.36	0.932
	Saccade Mean†	0.021	8.98 ± 3.04	8.95 ± 2.82	0.830
	Saccade Degree SD†	0.022	29.10 ± 8.24	29.41 ± 7.78	0.630
	Saccade Degree Total†	0.021	7808.41 ± 3444.85	7217.25 ± 3230.01	0.233
	Fixation Time Max†	0.020	26.81 ± 4.87	27.97 ± 4.08	0.213
Anti-saccade task	Saccade Degree Mean	0.033	42.92 ± 5.98	40.82 ± 8.63	0.119
	Saccade Duration	0.029	52.44 ± 13.73	48.18 ± 18.77	0.151
	Saccade Degree Total†	0.028	20869.22 ± 7520.73	18612.54 ± 8268.31	0.061
	Fixation Duration†	0.024	104.47 ± 33.88	103.06 ± 30.69	0.918
	Coordinate Y Mean	0.025	20.20 ± 94.26	10.44 ± 108.63	0.588
	Saccade Degree SD	0.028	35.70 ± 8.52	35.76 ± 8.43	0.967
	Fixation Count	0.019	1131.79 ± 236.06	1168.73 ± 241.93	0.379
	Screen Duration Mean†	0.023	3.77 ± 4.91	3.18 ± 3.51	0.768
	Saccade Latency SD†	0.020	0.85 ± 0.36	0.93 ± 0.62	0.520
	Saccade Time Max	0.025	24.68 ± 6.58	22.19 ± 7.41	0.047
	Saccade Latency Total†	0.020	76.85 ± 13.23	77.54 ± 17.02	0.799
Memory-guided saccade task	Saccade Degree Total†	0.048	14201.95 ± 6935.07	10647.83 ± 4475.26	0.005
	Saccade Degree Mean†	0.033	40.88 ± 6.05	37.48 ± 8.45	0.032
	Saccade Time Max†	0.035	17.91 ± 6.64	14.96 ± 5.47	0.007
	Fixation Time Max†	0.036	32.41 ± 7.17	35.54 ± 4.78	0.020
	Fixation Mean†	0.039	28.71 ± 8.09	32.27 ± 6.36	0.013
	Saccade Latency Total	0.033	51.14 ± 12.20	55.75 ± 10.84	0.022
	Saccade Duration†	0.029	42.41 ± 17.58	35.11 ± 14.18	0.011
	Coordinate X Mean†	0.023	1.78 ± 88.28	-13.09 ± 90.57	0.198
Change detection task	Saccade Degree SD	0.057	24.67 ± 4.76	27.47 ± 6.61	0.008
	Total Elapsed Time†	0.038	5658.91 ± 1333.01	5172.72 ± 1088.88	0.051
	Saccade Degree Mean	0.024	34.38 ± 7.12	34.06 ± 7.25	0.805
	Saccade Time Max†	0.025	12.36 ± 5.08	13.27 ± 5.03	0.219
Stroop task	Saccade Degree Mean†	0.052	33.67 ± 6.43	30.54 ± 6.35	0.003
	Screen Duration Mean†	0.037	0.42 ± 0.32	0.33 ± 0.34	0.019
	Saccade Latency Total†	0.043	2.66 ± 0.75	2.33 ± 0.65	0.012

Features are sorted by importance rank in the recursive feature elimination process.

Feature importance was calculated by an averaged importance from the classification analysis using an optimal model that includes all 33 eye-tracking features.

ADHD, Attention-Deficit/Hyperactivity Disorder; TDC, Typically Developing Children.

†Mann-Whitney U test was applied because of violations of the normality assumption.

**Supplementary Table 4** summarizes the classification metrics of each ML model in selecting the ADHD group using the optimal eye-tracking features model. The accuracies of the two models were greater than that of the Dummy classifier (accuracy of 0.583). The ‘RandomForest’ algorithm was found to have the highest accuracy (0.705), followed by the ‘ExtraTrees’ classifier algorithm (0.687).

**Figure 3:**
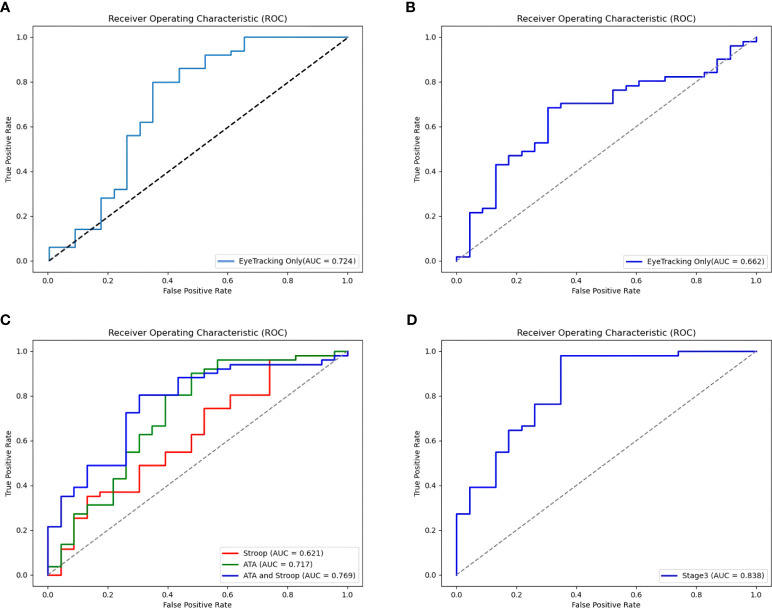
Receiver Operating Characteristic (ROC) curves for the models of ADHD by Ensemble stage. **(A)** An ROC curve for eye-tracking-features-only model in a large sample (135 subjects). **(B)** An ROC curve for Stage 1: Eye-tracking features only (73 subjects). **(C)** ROC curves for Stage 2: Behavioral task features (Features from the Visual and Auditory ATA, the Stroop task, and both ATAs and Stroop task). **(D)** An ROC curve for Stage 3: Ensemble of eye-tracking features, behavioral task feature (Features from the Stroop, and the visual ATA), demographics (age, gender, IQ), and clinical characteristics (CBCL, DBDRS, CDI, BDI, SCARED and FACES-IV).

The final soft voting model combining the RandomForest and ExtraTrees classifiers showed an accuracy of 76.3% and good AUC (0.724), recall (0.725), precision (0.789), and F1-score (0.789) among the 135 participants (**Figure 3A**).

### Comparison of the performance of models using eye-tracking features only versus models using behavioral, demographic, and clinical features

Multidimensional feature combination was performed using valid data from 73 participants. The eye-tracking-features-only model correctly classified 68.9% of diagnoses in stage 1 (**Table 4**). Compared to stage 1, the model using the Stroop task features had a better accuracy of 73.0%, but a more modest AUC of 0.621 (**Figure 3B**). The accuracy of the models increased to 78.4% and 77.0% when using the visual and auditory ATA features, and when using both ATAs and the Stroop task features, respectively. The AUC was the highest for the combination of both ATAs and the Stroop task features (0.769), but no significant differences were found between the stage 2 model and the eye-tracking-features-only model (**Figure 3C**, both ATAs vs. stage 1, U= 2549, p =0.235; Stroop task vs. stage 1, U= 2684, p =0.419; both ATAs and Stroop task vs. stage 1, U= 2524, p =0.206).

**Table 4:** Classification performance of the ensemble model of the multi-dimensional feature.

Model	Accuracy	Recall	Precision	F1-score	AUC	Comparison with Stage 1 (p-value)
Stage 1						
Eye-tracking feature only	0.689	0.961	0.700	0.810	0.662	–
Stage 2						
Stroop task	0.730	0.980	0.725	0.833	0.621	0.419
Visual and auditory ATA	0.784	0.961	0.778	0.860	0.717	0.235
Both ATAs and Stroop task	0.770	0.941	0.774	0.850	0.769	0.206
Stage 3						
Ensemble of eye-tracking, demographic, behavioral, clinical features	0.865	0.980	0.847	0.909	0.838	0.208

AUC, Area under the curve; ATA, Advanced test of attention.

In stage 3, the classification accuracy was the highest among all stages (86.5%), while the other ML model metrics were also improved. However, a comparison of ROC curves demonstrated no significant differences between the AUCs of the stage 3 and stage 1 models (**Figure 3D**, U= 2525, p =0.208).

## Discussion

The current study revealed that a classification model using eye-tracking features was useful in differentiating between children with ADHD and TDC. Although the age and IQ of the ADHD group were similar to those of the TDC group, the ML model using eye-tracking features could only successfully classify the diagnosis of the participants with an accuracy of 76.3%. An ensemble of demographic, behavioral, and clinical features increased the accuracy and precision metrics of the model, resulting in the correct classification of diagnosis. However, there was no significant difference in the AUC among the ensemble models.

Two tree-based decision algorithms were effective at classifying ADHD. The optimal number of features for classification varied for each task; some showed significant differences in conventional statistics, whereas others did not. In decision tree analysis, each feature is assigned a weight (importance) based on its attributes for classification at each splitting step (44). Decision tree integrates weights from various features to improve overall performance compared to a single classifier. Because of these characteristics, individual classifiers tend to make errors under different circumstances (45). Thus, some of the eye-tracking features selected in the optimal model may not be significant in conventional statistical analysis. In each task, the tree-based algorithm tended to include more features in the model when the difference in eye-tracking features between the two groups was not distinct (i.e., PST and AST), and to classify with fewer features when there were significant differences (i.e., MGST, CDT, and Stroop task). This finding suggests that features in the MGST, CDT, and the Stroop task may be sensitive indicators for classifying ADHD.

In the current study, a total of 33 features useful for ADHD classification were chosen from five eye-tracking tasks. We examined the role of features in each task in relation to attentional control, working memory, and response inhibition in ADHD.

The neural mechanisms of saccadic eye movements are closely linked to those of attentional control (11, 46). Munoz et al. (9) demonstrated that individuals with ADHD exhibited extended RT, higher within-subject variability, and prolonged durations during the PST. A recent review also showed that the mean latency of pro-saccades in ADHD was significantly shorter, but the error rate was higher than controls in multiple studies (10, 47). The ADHD group also showed a reduced ability to suppress involuntary saccades when asked to fix their gaze (9, 12, 47). Similarly, we found that participants with ADHD showed significantly shorter fixation durations in the PST than the TDC. The ADHD group also tended to have longer saccade latencies and greater saccade degrees, but no statistically significant differences were found. These results suggest that individuals with ADHD are associated with reduced fixation time and shorter saccade latency in the PST, which has been implicated in poor attentional control.

The mechanisms underlying AST are thought to be associated with top-down inhibition of saccade-generating neurons (48). The ADHD group was found to have more errors in the task, as well as greater RT and within-subject variability (9). They also showed longer RT and more anti-saccade direction errors than TDCs (47, 49). Another report demonstrated that ADHD children had a lower ability to suppress explorative saccade than controls (50). In accordance with the aforementioned findings, this study observed a significant increase in the longest time spent on saccades (Saccade Time Max) during the AST in the ADHD group compared to the TDC group. In addition, ADHD children showed a larger change in the Y-axis coordinate as well as an increase in saccade degree total and saccade degree mean compared to controls. This result indicates that the ADHD group had difficulty inhibiting the saccade responses, as evidenced by their failure to interrupt the provocative saccade of visual stimuli.

The MGST (36, 37) and the CDT (38, 39) were both designed to reveal deficits in visuospatial working memory among participants with ADHD. In individuals with ADHD, the decreased accuracy, higher level of anticipatory errors, and prolonged latencies in MGS have been reported (22, 51). Hence, it has been reported that the ADHD group did not show a significant difference in performance time compared to controls (38), but were less accurate in the CDT. This may occur due to impairments in response inhibition, and/or deficits in visuospatial working memory (16). In parallel with these findings, we observed that the ADHD group had a wider range of gaze movements, relatively shorter fixation times, and more saccadic eye movements during the MGST. In the CDT, individuals with ADHD showed large variation in Saccade Degree (Saccade Degree SD) which is consistent with recent literature (52, 53).

Finally, in the Stroop task, the ADHD group showed a significant increase in saccade degree and latency compared with the TDC group. Previous studies have shown that features of ADHD include more frequent gaze switches between the target and distractor, a higher overall time spent on the target and distractor, and a higher number of fixations on the target (41). Our experiments could not measure the gaze switch itself; however, repeated saccade movement and hesitation before choosing the correct answer in the ADHD group may be associated with a greater saccade degree and saccade latency in need of decision making whether a stimulus is congruent or not. Although we did not identify any significant differences in accuracy in the Stroop task, working memory and response inhibition presumably influenced the time taken to make a decision in the Stroop interference (40).

The primary strength of this study is that we were able to distinguish the ADHD and TDC groups using only eye-tracking features. Compared to conventional screening methods, such as the ATA or Stroop task, the eye-tracking-features-only model was slightly less accurate, but performed equally well on recall metrics, and did not show a significant difference in AUC. The ensemble of eye tracking, demographic, behavioral, and clinical data showed the best accuracy among all models, but there was no significant difference in the AUC compared with the stage 1 model. Another important point is that a simple smart pad device can be used to obtain eye-tracking data. Smart pad devices are commonly used in many households, and it is expected that the use of currently developed eye-tracking algorithms can be easily applied to reduce the temporal and spatial constraints associated with screening for ADHD.

Nevertheless, this study has several limitations which should be considered. Firstly, the sample size was small in the group that completed the behavioral tasks and provided clinical data. This limit would lead to trade-off between model complexity and accuracy and may have caused overfitting problems in the ML analysis. However, we used a tree-based algorithm to overcome this overfitting by setting the node depth to reduce overfitting, and subsequently found that the eye-tracking features alone could significantly classify ADHD. Second, in terms of sex, there were significantly more boys than girls in the ADHD group, but not in the TDC group. In the future, it may be necessary to conduct a sex-matched study to overcome this effect. Finally, the accuracy of eye-tracking metrics could have been affected by the children’s head movements. In this study, we attempted to improve this accuracy by excluding a large amount of inaccurate data, but we also believe that higher-quality eye-tracking data can be obtained using a virtual reality (VR) head-mounted eye-tracking system.

In conclusion, our optimal model successfully classified ADHD by combining different eye-tracking features extracted from behavioral tasks that reflect the core problems of ADHD, such as difficulty in selective attention, working memory, and response inhibition. Additionally, the present study is expected to be highly applicable to ADHD screening as it measures eye-tracking features using a simple digital device. Future research will need to improve the measurement methods, such as using a VR headset, and further elaborate on model accuracy by including more participants and matching demographic and clinical characteristics.

## Data availability statement

The original contributions presented in the study are included in the article/**Supplementary Material**. Further inquiries can be directed to the corresponding author.

## Ethics statement

The studies involving humans were approved by Institutional Review Boards of Seoul National University (SNU) Hospital (Approval Number, 2103-197-1208), Institutional Review Boards of Chung-Ang University Hospital (2160-003-464), Institutional Review Boards of Seoul St. Mary’s Hospital (KC21FIDI0355), Institutional Review Boards of Seoul Metropolitan Government (SMG)–SNU Boramae Medical Center (30-2021-111). The studies were conducted in accordance with the local legislation and institutional requirements. Written informed consent for participation in this study was provided by the participants’ legal guardians/next of kin.

## Author contributions

JY: Conceptualization, Data curation, Formal analysis, Investigation, Writing – original draft. CK: Conceptualization, Formal analysis, Investigation, Writing – original draft. JL: Conceptualization, Formal analysis, Supervision, Writing – review & editing. BW: Conceptualization, Formal analysis, Supervision, Writing – review & editing. C-HC: Data curation, Investigation, Writing – review & editing. HH: Data curation, Investigation, Writing – review & editing. DH: Conceptualization, Data curation, Investigation, Writing – review & editing. HK: Conceptualization, Funding acquisition, Resources, Software, Validation, Writing – review & editing. HC: Conceptualization, Funding acquisition, Resources, Software, Validation, Writing – review & editing. J-WK: Conceptualization, Data curation, Investigation, Project administration, Supervision, Validation, Writing – review & editing.
