# Supplementary material for: Development of an innovative approach using portable eye tracking to assist ADHD screening: a machine learning study
Source: Front Psychiatry. 2024 Feb 15;15:1337595. doi: 10.3389/fpsyt.2024.1337595 (PMC10902460; doi:10.3389/fpsyt.2024.1337595)
Supplement: Supplementary file 1 [file DataSheet_1.pdf]

Table S1. Clinical and behavioral characteristics of the sub-group participants (n=73)

Variables	ADHD (n=50)	TDC (n=23)	P value
Age, years†	8.48 ± 1.63	9.04 ± 1.77	.239
Gender (M:F)	39:11	12:11	**0.025**
IQ	104.74 ± 17.40	106.04 ± 18.23	.770
ADHD-RS total score	25.98 ± 7.81	9.91 ± 6.59	**<0.001**
ADHD-RS inattention score	15.00 ± 4.58	6.09 ± 4.10	**<0.001**
ADHD-RS hyperactivity /impulsivity score	10.98 ± 4.99	3.83 ± 3.26	**<0.001**
ATA visual, OE†	76.40 ± 22.33	62.57 ± 16.49	**0.017**
ATA visual, FA†	73.28 ± 20.35	62.13 ± 21.59	**0.022**
ATA visual, RT	62.98 ± 14.37	64.52 ± 9.92	0.643
ATA visual, RTV†	68.84 ± 20.53	63.22 ± 17.07	0.360
ATA Auditory, OE†	71.44 ± 21.54	58.26 ± 19.65	**0.005**
ATA Auditory, FA†	72.64 ± 20.67	58.35 ± 18.48	**0.004**
ATA Auditory, RT†	46.34 ± 15.04	57.70 ± 8.13	**<0.001**
ATA Auditory, RTV	53.28 ± 14.87	45.91 ± 10.18	**0.008**
Stroop word	38.60 ± 10.93	44.26 ± 8.87	**0.033**
Stroop color	43.28 ± 11.48	47.22 ± 7.65	0.089
Stroop color word	45.28 ± 12.06	51.57 ± 10.13	**0.033**
Stroop Interference	53.66 ± 12.05	55.13 ± 8.92	0.603
CDI†	11.24 ± 8.44	7.26 ± 4.40	**0.024**
BDI†	8.78 ± 11.01	3.52 ± 4.59	**0.002**
SCARED, total score†	20.32 ± 15.14	13.78 ± 6.99	0.087
SCARED, panic score†	3.30 ± 5.06	1.52 ± 1.41	0.281
SCARED, general anxiety score†	4.58 ± 4.28	2.74 ± 2.65	0.081
SCARED, separation anxiety score†	6.42 ± 3.75	5.13 ± 3.82	0.154
SCARED, social anxiety score†	4.86 ± 3.53	3.91 ± 2.84	0.269
SCARED, school avoidance score†	1.16 ± 1.98	0.48 ± 0.73	0.334
FACES-IV, total score	47.60 ± 12.47	52.35 ± 10.94	0.121
FACES-IV, adaptability score†	19.52 ± 6.78	21.96 ± 5.51	0.070
FACES-IV, cohesion score	28.08 ± 6.73	30.39 ± 6.44	0.171
CBCL, Total, score	56.52 ± 8.56	44.91 ± 7.73	**<0.001**
CBCL, Internalizing score†	53.58 ± 11.26	46.70 ± 7.30	**0.015**
CBCL, Externalizing score	55.72 ± 8.92	45.65 ± 9.07	**<0.001**
CBCL, Withdrawn†	55.36 ± 10.86	47.61 ± 6.77	**0.002**
CBCL, Somatic complaints†	49.64 ± 9.65	47.30 ± 6.78	0.469
CBCL, Anxious/Depressed†	53.48 ± 11.72	46.87 ± 8.96	**0.024**
CBCL, Social problems†	55.26 ± 11.86	43.70 ± 6.95	**<0.001**
CBCL, Thought problems†	55.12 ± 9.93	49.17 ± 5.84	**0.013**
CBCL, Attention	59.22 ± 7.79	45.13 ± 8.15	**<0.001**
CBCL, Delinquent behavior†	51.52 ± 9.40	44.47 ± 8.01	**0.002**
CBCL, Aggressive behaviors	56.40 ± 9.31	46.30 ± 8.89	**<0.001**
CBCL, Sex problems†	51.78 ± 8.26	48.52 ± 5.73	0.099
CBCL, Emotional problems†	48.74 ± 9.06	42.17 ± 7.52	**0.004**
DBDRS, Total, score†	8.02 ± 6.06	3.17 ± 3.39	**<0.001**
DBDRS, ODD score†	6.86 ± 4.90	2.96 ± 3.17	**<0.001**
DBDRS, CD score†	1.16 ± 1.77	0.22 ± 0.52	**0.012**

† Mann-Whitney U test was applied because of violations of the normality assumption.

ADHD, Attention-Deficit/Hyperacitivity Disorder; TDC, Typically Developing Children; ADHD-RS, Dupaul's

ADHD rating Scale, Korean version; ATA, Advanced test of Attention; OE, Omission Error; FA, False Alaram; RT, Reaction Time; RTV, Reaction Time Variability; CDI, Children's depression Inventory; BDI, Beck Depression Inventory; SCARED, Screen for Child Anxiety Related Disorders; FACES-IV, Family Adaptability and Cohesion Scale IV; CBCL, Child Behavior Checklist, DBDRS, Disruptive Behaviour Disorder Rating Scale; ODD, Oppostional Defiant Disroder; CD, Conduct Disorder

Table S2. Correlation analysis results between eye-tracking feature and ADHD-RS, Stroop task parameters

Task sequence	Feature	ADHD-RS-I	ADHD-RS-H	ADHD-RS-T	SCWT-W	SCWT-C	SCWT-CW	SCWT-I
Pro-saccade task	Fixation Duration	-0.02	0.04	0.01	-0.02	-0.11	-0.20	-0.05
	Saccade Degree Mean	0.04	0.14	0.10	-0.20	-0.09	-0.08	0.01
	Saccade Latency Mean	0.10	0.01	0.06	-0.12	-0.11	-0.08	0.01
	Saccade Mean	-0.10	-0.02	-0.06	0.09	0.15	0.11	-0.04
	Saccade Degree SD	-0.17*	-0.08	-0.14	-0.08	0.04	0.00	-0.07
	Saccade Degree Total	-0.04	0.05	0.00	0.01	0.09	0.05	-0.05
	Fixation Time Max	0.00	0.06	0.03	-0.10	-0.15	-0.16	0.01
Anti-saccade task	Saccade Degree Mean	0.07	0.09	0.09	-0.07	-0.01	-0.13	-0.12
	Saccade Duration	-0.04	0.00	-0.02	0.02	0.11	0.17	0.01
	Saccade Degree Total	0.04	0.01	0.03	-0.05	0.07	0.09	0.01
	Fixation Duration	-0.05	0.15	0.05	0.02	-0.07	-0.16	-0.14
	Coordinate Y Mean	0.08	-0.08	0.00	-0.14	-0.06	0.09	0.17
	Saccade Degree SD	0.03	-0.02	0.01	-0.07	-0.01	0.07	0.10
	Fixation Count	-0.09	0.07	-0.02	0.05	-0.06	-0.24*	-0.22
	Screen Duration Mean	0.09	-0.04	0.03	-0.16	-0.03	0.11	0.13
	Saccade Latency SD	0.10	0.03	0.07	-0.14	-0.21	-0.10	0.18
	Saccade Time Max	0.05	0.00	0.03	-0.06	0.08	0.20	0.12
	Saccade Latency Total	0.02	0.02	0.02	0.03	-0.07	-0.27*	-0.22
Memory-guided saccade task	Saccade Degree Total	0.09	0.11	0.11	0.16	0.16	0.20	0.00
	Saccade Degree Mean	0.09	0.17	0.13	0.01	0.09	0.14	0.02
	Saccade Time Max	0.04	0.04	0.04	0.20	0.22*	0.21	-0.06
	Fixation Time Max	-0.08	-0.03	-0.06	-0.01	-0.12	-0.20	-0.07
	Fixation Mean	-0.11	-0.02	-0.08	0.01	-0.11	-0.18	-0.07
	Saccade Latency Total	-0.12	-0.13	-0.13	-0.09	-0.20	-0.22*	0.01
	Saccade Duration	0.00	-0.02	-0.01	0.20	0.15	0.17	0.00
	Coordinate X Mean	0.16	0.06	0.12	-0.07	0.00	-0.03	-0.02
Change detection task	Saccade Degree SD	-0.10	-0.03	-0.07	-0.03	-0.04	-0.06	0.02
	Total Elapsed Time	0.16	0.21*	0.20*	-0.09	0.05	0.09	0.00
	Saccade Degree Mean	-0.09	-0.02	-0.06	-0.02	-0.03	-0.03	0.01
	Saccade Time Max	-0.17*	-0.14	-0.17*	0.21	0.04	-0.08	-0.13
Stroop task	Saccade Degree Mean	0.16	0.15	0.17	-0.18	-0.10	-0.04	0.07
	Screen Duration Mean	0.01	0.00	0.01	-0.18	0.05	0.14	0.07
	Saccade Latency Total	0.11	0.15	0.14	-0.03	0.00	0.05	0.07

*p<0.05

Features are sorted by importance rank in recursive feature elimination process

ADHD, Attention-Deficit/Hyperacitivity Disorder; TDC, Typically Developing Children

Table S3. Correlation analysis results between eye-tracking feature and ATA task parameters

Task sequence	Feature	ATA-V-OE	ATA-V-FA	ATA-V-RT	ATA-V-RTV	ATA-A-OE	ATA-A-FA	ATA-A-RT	ATA-A-RTV
Pro-saccade task	Fixation Duration	0.05	0.02	-0.03	0.06	-0.08	0.10	-0.15	0.02
	Saccade Degree Mean	0.21	0.00	0.15	0.08	0.09	0.09	-0.11	0.10
	Saccade Latency Mean	-0.09	-0.01	-0.08	-0.05	0.02	-0.06	0.02	-0.12
	Saccade Mean	-0.04	0.00	0.05	-0.12	-0.13	-0.04	0.02	0.01
	Saccade Degree SD	0.02	0.04	0.08	-0.08	-0.01	-0.08	0.13	0.12
	Saccade Degree Total	0.08	0.06	0.10	-0.02	-0.08	0.03	-0.02	0.09
	Fixation Time Max	0.14	0.05	-0.04	0.19	0.07	0.16	-0.27*	-0.03
Anti-saccade task	Saccade Degree Mean	0.08	-0.08	0.11	0.01	-0.12	0.07	0.01	0.06
	Saccade Duration	0.07	0.01	0.06	-0.09	-0.09	-0.01	-0.03	0.05
	Saccade Degree Total	0.02	-0.08	0.09	-0.15	-0.13	-0.01	0.10	0.12
	Fixation Duration	0.07	0.09	-0.05	0.12	-0.01	0.01	-0.21	-0.16
	Coordinate Y Mean	-0.24*	-0.19	-0.06	-0.16	0.05	-0.07	0.07	-0.08
	Saccade Degree SD	0.05	-0.04	0.06	-0.05	-0.10	0.00	0.21	0.22
	Fixation Count	0.03	0.00	-0.03	0.08	-0.01	0.05	-0.17	-0.14
	Screen Duration Mean	0.01	-0.06	0.13	-0.05	-0.14	-0.03	0.14	0.17
	Saccade Latency SD	0.14	0.21	-0.09	0.31**	0.13	0.05	-0.15	0.08
	Saccade Time Max	0.10	-0.07	0.11	-0.12	-0.08	-0.02	0.09	0.11
	Saccade Latency Total	0.00	0.07	-0.07	0.17	0.06	0.19	-0.10	-0.03
Memory-guided saccade task	Saccade Degree Total	0.00	0.01	-0.06	-0.12	-0.08	0.01	0.00	0.00
	Saccade Degree Mean	0.05	-0.07	0.05	-0.02	-0.12	0.00	-0.02	0.00
	Saccade Time Max	0.04	0.05	-0.10	-0.10	-0.03	0.04	-0.02	0.03
	Fixation Time Max	0.00	-0.09	0.04	0.15	0.09	0.06	-0.10	-0.10
	Fixation Mean	-0.02	-0.06	0.02	0.17	0.05	0.07	-0.13	-0.12
	Saccade Latency Total	-0.06	-0.17	0.12	0.14	0.11	0.14	-0.04	0.03
	Saccade Duration	0.03	0.05	-0.11	-0.10	-0.06	0.05	-0.02	-0.04
	Coordinate X Mean	0.02	0.01	-0.07	-0.12	-0.10	-0.18	0.07	-0.09
Change detection task	Saccade Degree SD	0.08	-0.03	-0.08	-0.03	0.12	0.08	0.01	0.06
	Total Elapsed Time	-0.04	-0.09	0.13	-0.11	0.06	-0.03	0.04	0.18
	Saccade Degree Mean	0.14	0.01	-0.03	0.08	0.04	0.11	-0.06	0.03
	Saccade Time Max	0.12	0.05	-0.18	0.02	-0.07	0.00	-0.07	-0.26*
Stroop task	Saccade Degree Mean	0.23*	0.16	-0.05	0.15	0.09	0.18	-0.17	0.07
	Screen Duration Mean	0.03	-0.08	0.12	0.00	-0.01	-0.11	0.10	-0.09
	Saccade Latency Total	0.06	0.00	0.16	0.08	-0.10	0.03	0.05	0.17

Features are sorted by importance rank in recursive feature elimination process

ADHD, Attention-Deficit/Hyperacitivity Disorder; TDC, Typically Developing Children

Table S4. Prediction performance of the eye-tracking feature only model among various ML algorithms

Model	Accuracy	Recall	Precision	F1-score	AUC
RandomForest Classifier	0.705	0.444	0.745	0.551	0.710
Extra Trees Classifier	0.687	0.489	0.696	0.562	0.702
Naïve Bayes	0.667	0.467	0.651	0.536	0.703
Extreme Gradient Boosting	0.649	0.533	0.588	0.557	0.733
CatBoost Classifier	0.641	0.400	0.610	0.474	0.724
Quadratic Discriminant Analysis	0.611	0.067	0.400	0.113	0.601
K Neighbors Classifier	0.595	0.400	0.516	0.448	0.629
Linear Discriminant Analysis	0.594	0.489	0.516	0.493	0.587
Ridge Classifier	0.584	0.422	0.487	0.446	0.000
Dummy Classifier	0.583	0.000	0.000	0.000	0.500
Gradient Boosting Classifier	0.576	0.400	0.481	0.436	0.690
Light Gradient Boosting Machine	0.575	0.422	0.470	0.435	0.698
Logistic Regression	0.575	0.422	0.481	0.445	0.628
Decision Tree Classifier	0.575	0.467	0.455	0.457	0.558
Ada Boost Classifier	0.538	0.356	0.464	0.390	0.633
SVM – Linear Kernel	0.537	0.489	0.449	0.468	0.000

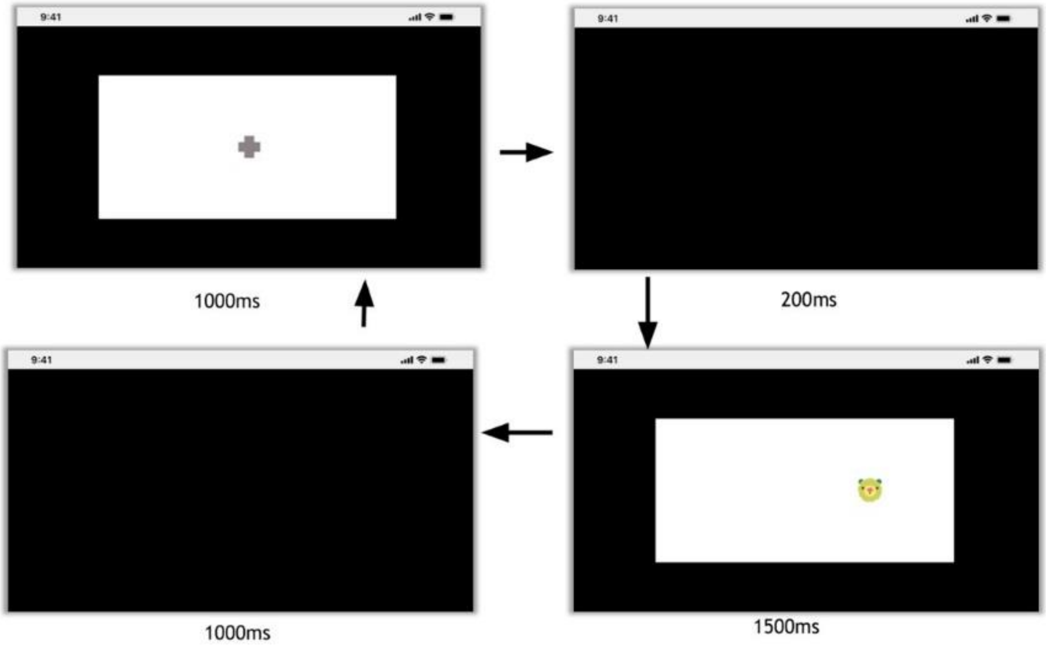

Figure S1. The procedure of the pro-saccade task (PST)

Participants first focused on a central fixation point (FP) and then shifted their gaze to a peripheral target stimulus that appeared unpredictably on the left or right side of the Smart Pad device. After 1,000 ms, the FP disappeared, followed by the appearance of an eccentric target stimulus that was illuminated for 1,000 ms.

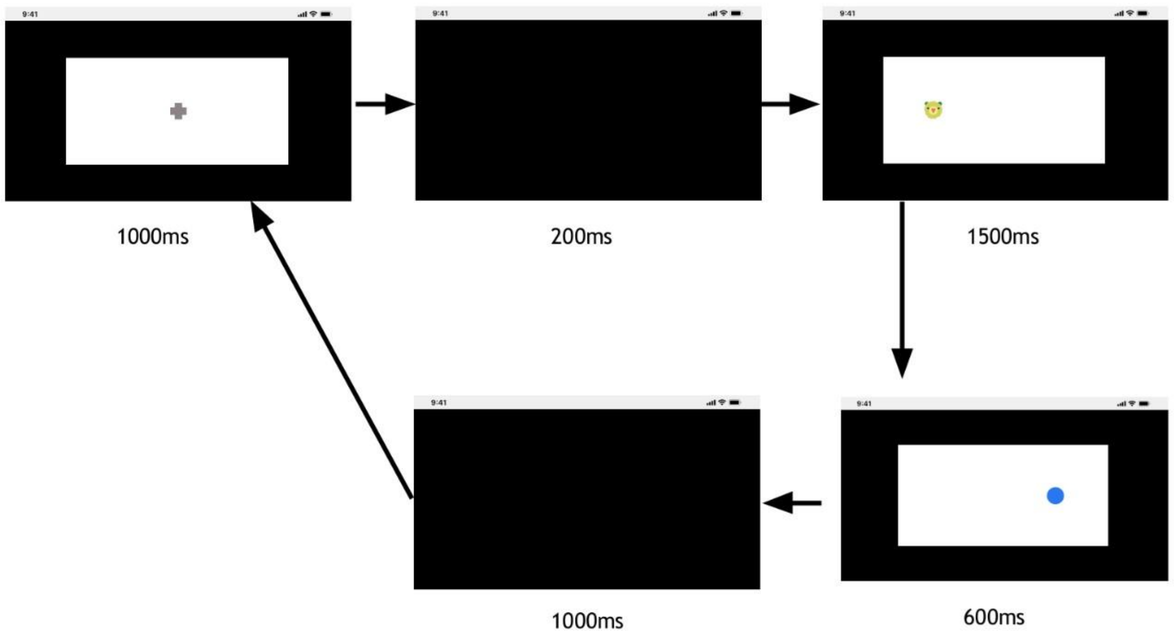

Figure S2. The procedure of the anti-saccade task (AST)

Participants directed their gaze to the central FP and, upon appearance of the eccentric stimulus, shifted their gaze away from it to the opposite side of the vertical meridian. This anti-saccade task consisted of 48 trials, 5 s apart.

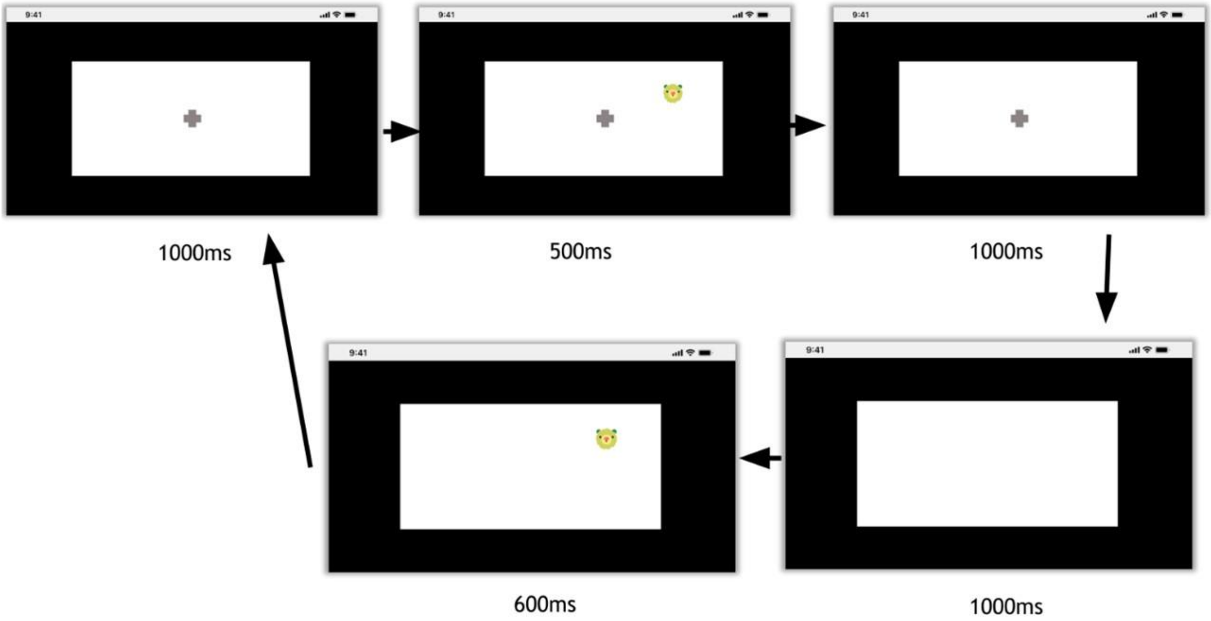

Figure S3. The procedure of the Memory-guided saccade task (MGST)

Participants first focused on the FP for 1,000 ms before a target stimulus briefly appeared in the peripheral region of the screen (500 ms). After the FP disappeared, participants had to remember the location and move their gaze to it in the absence of a visual cue. Stimulus locations were randomized across trials in the 36-trial.

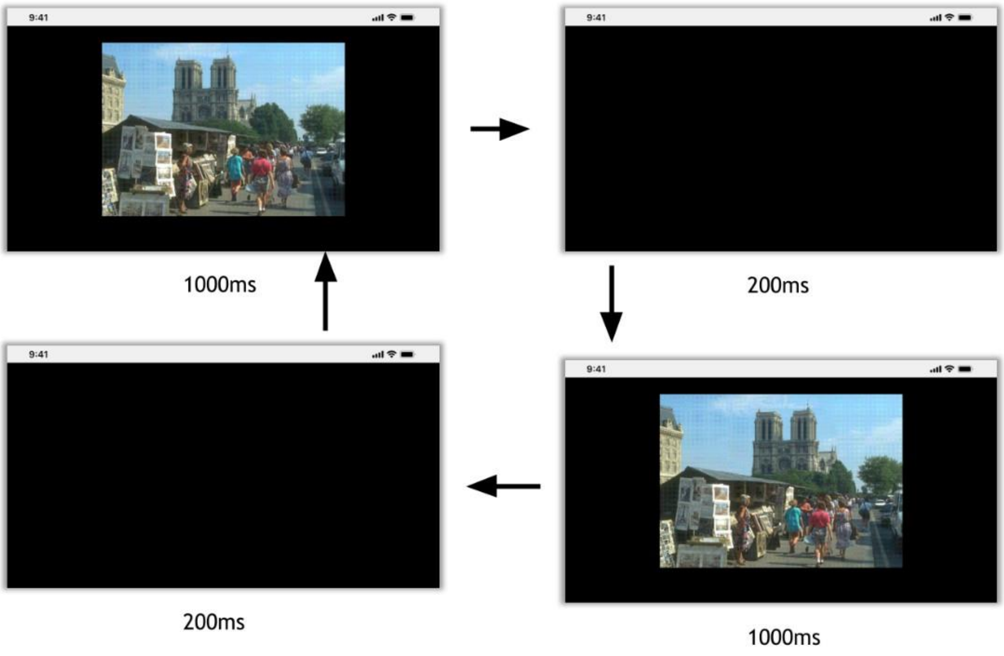

Figure S4. The procedure of the Change detection task (CDT)

The CDT consisted of 15 pairs of images presented sequentially with small differences. Participants had 240 ms to identify differences by touching the location they judged to be different and then moving on to the next pair, with incorrect responses moving on to the next pair after 20 seconds.

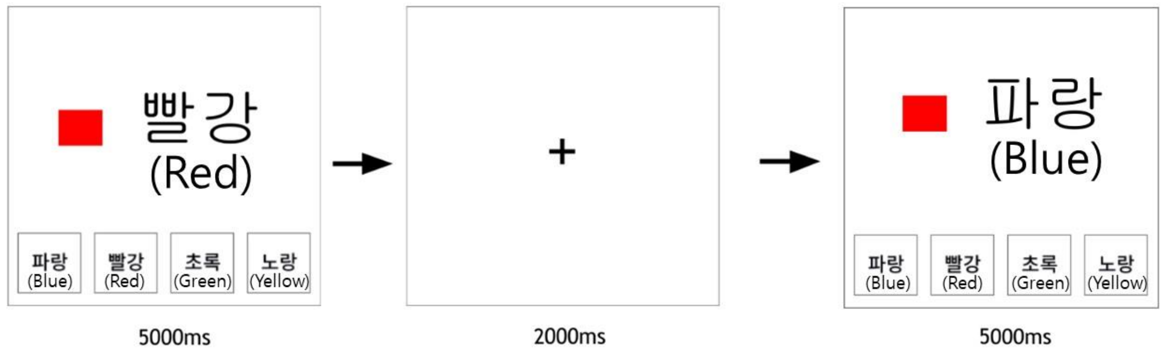

Figure S5. The procedure of the Stroop task

Participants was asked to identify colors presented in rectangles with corresponding color names. They completed 40 trials, discriminating between congruent and incongruent stimuli within 5,000 ms, followed by 2,000 ms of FP indicating trial changes. Incorrect responses led them to the next trial after 5 seconds.
